# Supplementary material for: Exploring Genome-Wide Mutation Dynamics and Bacterial Cellulose Impairment in Komagataeibacter intermedius Cultivated Under Agitation Stress
Source: Comput Struct Biotechnol J. 2026 Mar 18;35(1):0009. doi: 10.34133/csbj.0009 (PMC13068021; doi:10.34133/csbj.0009)
Supplement: Supplementary 1 — Additional File 1 Figs. S1 to S12 Tables S1 and S2 Additional Files 2 to 5 [file csbj.0009.f1.zip › Revised_Additional File 1_Clean_CSBJ-D-25-01794.docx]

SUPPLEMENTARY MATERIALS _ Additional file 1

**Exploring Genome-Wide Mutation Dynamics and Bacterial Cellulose Impairment in *Komagataeibacter intermedius* Cultivated Under Agitation Stress**

Davide Bersanetti, Rahul Mangayil

Department of Bioproducts and Biosystems, Aalto University, Espoo, Finland.

[davide.bersanetti@aalto.fi](mailto:davide.bersanetti@aalto.fi) +393476590846

[rahul.mangayil@aalto.fi](mailto:rahul.mangayil@aalto.fi); +358509114514

OrCiD

Davide Bersanetti 0009-0001-9987-4220

Rahul Mangayil 0000-0001-7547-409X

# **Additional Figures**

A

**5**

**4**

**3**

**2**

**1**

**10**

**9**

**8**

**7**

**6**

B

**5**

**4**

**3**

**2**

**1**

**10**

**6**

**9**

**8**

**7**

C

**5**

**4**

**2**

**3**

**1**

**10**

**9**

**8**

**7**

**6**

Figure 1: BC synthesis and planktonic growth of K. ENS15 lineages under agitated cultivation. A) Flask A over ten rounds (1–10), showing complete loss of cellulose production by Round 4. B) Flask B over ten rounds (1–10), similarly exhibiting no cellulose production starting at Round 4. C) Flask C over ten rounds (1–10), with cellulose clearly produced until round 5 but completely halted from Round 6 onward

Flask A

OD 600

| Round number | Flask A |
| --- | --- |
| 1 | 2 |
| 2 | 2.2 |
| 3 | 2.1 |
| 4 | 3.3 |
| 5 | 2.46 |
| 6 | 2.48 |
| 7 | 1.87 |
| 8 | 2 |
| 9 | 1.06 |
| 10 | 2.16 |

A

Figure 2. Cell growth (OD_600nm_) measurement across 10 cultivation rounds for all three cultivation lines. A) represent the OD600nm measurement for Flask A. B) represent the OD600nm measurement for Flask B. C) represent the OD600nm measurement for Flask C

Flask B

| Round number | OD 600 |
| --- | --- |
| 1 | 2 |
| 2 | 1.94 |
| 3 | 2 |
| 4 | 1.85 |
| 5 | 2.16 |
| 6 | 1.76 |
| 7 | 2 |
| 8 | 2.6 |
| 9 | 1.14 |
| 10 | 1.64 |

Flask C

| Round number | OD 600 |
| --- | --- |
| 1 | 2.5 |
| 2 | 2.1 |
| 3 | 2 |
| 4 | 2.4 |
| 5 | 2.64 |
| 6 | 2.64 |
| 7 | 2 |
| 8 | 2 |
| 9 | 1.06 |
| 10 | 2.12 |

B

C

A

BC static production from Flask A shaken cultivations

| Rounds | g L⁻¹ |
| --- | --- |
| 1 | 0.52 |
| 2 | 0.46 |
| 3 | 0.98 |
| 4 | 0.61 |
| 5 | 0.55 |
| 6 | 0.53 |
| 7 | 1.13 |
| 8 | 0.12 |
| 9 | 0.1 |
| 10 | 0.1 |

BC static production from Flask B shaken cultivations

| Rounds | g L⁻¹ |
| --- | --- |
| 1 | 0.63 |
| 2 | 0.36 |
| 3 | 0.55 |
| 4 | 0.31 |
| 5 | 0.18 |
| 6 | 0.88 |
| 7 | 1.27 |
| 8 | 0.05 |
| 9 | 0.36 |
| 10 | 0.28 |

B

BC static production from Flask C shaken cultivations

| Rounds | g L⁻¹ |
| --- | --- |
| 1 | 0.6 |
| 2 | 0.28 |
| 3 | 1.1 |
| 4 | 0.49 |
| 5 | 0.47 |
| 6 | 0.6 |
| 7 | 1.24 |
| 8 | 0.27 |
| 9 | 0.35 |
| 10 | 0.28 |

C

Figure 3. BC cellulose production (reported as g/L) achieved by static cultivations inoculated with cells obtained from the shaken cultivations. A) production achieved in Flask A. B) production achieved in Flask B. C) production achieved in Flask C.

Figure 4. Reads supporting a JC affecting the bcs operon IV in the parental strain. These reads, when analyzed with BLAST, match a transposase sequence.

Figure 5. Additional evidence supporting the JC affecting bcs operon IV in the parental strain. The reads covering the junction, when analysed by BLAST, show a clear association with transposase activity.

Figure 6. Translation of *ccpAx* made on Geneious. The image highlights the region containing 13 consecutive prolines, where replication slippages were detected in the parental strain; Flask A (rounds 4, 7, and 10); Flask B (rounds 2, 3, 5, 6, and 7); and Flask C (rounds 1, 2, and 10).

Figure 7. Translation, made on Geneious, of the *bcsA* with the 20 bp duplication that affected it in Flask C (rounds 9 and 10). The duplication leads to two premature stop codons.

C

B

A

Figure 8. 3D structures (made on SwissModel) of wild type *bcsC,* and the *bcsC* mutated by the 21 bp deletion that removes the ARFWLQQ amino acid sequence. A) The wild type *bcsC*. B) The mutated *bcsC*. C) superimposition of wild type bcsC 3D model on the mutant bcsC 3D model. Green colour indicate a match between the two models, while the red colour indicates a mismatch. For A, B,C the region affected by the mutation is circled in red.

Figure 9. Translation, made on Geneious, of the *bcsC* region following the 4 bp deletion detected in Flask A ( round5), Flask B ( rounds3,4,5) and Flask C (rounds5,6,7,8). The deletion cause multiple premature stop codons (shown in black).

Figure 10. Translation, made on Geneious, of the *bcsC* after the the duplication event (bp 1517909-928), which cause several premature stop codons ( in black) in Flask C, round 6.

Figure 11. Visualization, made on Geneious, of the deletion causing the loss of the start codon in the *pgm* gene, which is located on the reverse strand. The mutation was detected in Flask C, rounds 8,9,10.

Fluorescence values c-di-GMP assay Flask A pellets

| Round number | Fluorescence 458/528 |
| --- | --- |
| 1 | 552.5 |
| 2 | 717.6 |
| 3 | 584.25 |
| 4 | 950.9 |
| 5 | 656.04 |
| 6 | 564.6 |
| 7 | 546.7 |
| 8 | 1038.94 |
| 9 | 366.51 |
| 10 | 386.595 |

Figure 12. Fluorescence values from the intracellular c-di-GMP assay for pellets collected from Flask A after each cultivation round.

**Additional Tables**

| **Table 1.** Cultivation rounds in relation to the onset of planktonic growth and complete loss of bacterial cellulose (BC) across flasks | | |
| --- | --- | --- |
| **Flask** | **Onset of planktonic growth** | **BC loss** |
| A | Round 2 | Round 4 |
| B | Round 2 | Round 4 |
| C | Round 2 | Round 6 |

| Table 2. BLAST analysis conducted on putative PDE/DGC loci in K.ENS15, compared to the original annotation provided by Cannazza et al (20) | | |
| --- | --- | --- |
| Original annotation | **BLAST annotation** | **Genomic position** |
| dosP_1/Hypothetical protein | Putative bifucntional DGC_PDE / EAL-domain containing protein | 438431-442444 |
| dosP_2 | EAL domain containing protein | 768732-771215 |
| putative signaling protein / dosp_3 | Putative bifucntional DGC_PDE / EAL-domain containing protein | 935004-939007 |
| dosP_4 | EAL domain containing protein | 2393877-2396450 |
| hypothetical protein/hypothetical protein | EAL domain containing protein/ putative bifunctional DGC_PDE | 2400866-2404982 |
| dosP_5 | EAL domain containing protein | 2889745-2891967 |
| dosP_6 | Putative bifunctional DGC_PDE | 2892011-2894218 |
| hypothetical protein | EAL domain containing protein | 3602045-3604261 |
| dosP_7/putative signaling protein | EAL domain containing protein/ putative bifunctional DGC_PDE | 3604360-3608735 |

References

20.Cannazza P, Rissanen AJ, Sarlin E, Guizelini D, Minardi C, Losoi P, et al. Characterization, genome analysis and genetic tractability studies of a new nanocellulose producing Komagataeibacter intermedius isolate. Sci Rep. 2022 Nov 28;12(1):20520.
